# Field-Free Spin–Orbit Torque Switching of Canted van der Waals Magnets

**DOI:** 10.1021/acsnano.4c16826

**Published:** 2025-03-31

**Authors:** Bing Zhao, Lalit Pandey, Khadiza Ali, Erdi Wang, Craig M. Polley, Balasubramanian Thiagarajan, Peter Makk, Marcos H. D. Guimarães, Saroj Prasad Dash

**Affiliations:** † Department of Microtechnology and Nanoscience, Chalmers University of Technology, Göteborg SE-41296, Sweden; ‡ MAX IV Laboratory, Lund University, Lund SE-221 00, Sweden; § Department of Physics, Institute of Physics, Budapest University of Technology and Economics, Budapest H-1111, Hungary; ∥ Zernike Institute for Advanced Materials, University of Groningen, Groningen 9747AG, the Netherlands; ⊥ Wallenberg Initiative Materials Science for Sustainability, Department of Microtechnology and Nanoscience, Chalmers University of Technology, Göteborg SE-41296, Sweden; # Graphene Center, Chalmers University of Technology, Göteborg SE-41296, Sweden; ∇ MTA-BME Correlated van der Waals Structures Momentum Research Group, Műegyetem rkp. 3., Budapest H-1111, Hungary

**Keywords:** canted magnetization, spin−orbit torque, Fe_5_GeTe_2_, 2D magnets, 2D materials, room temperature

## Abstract

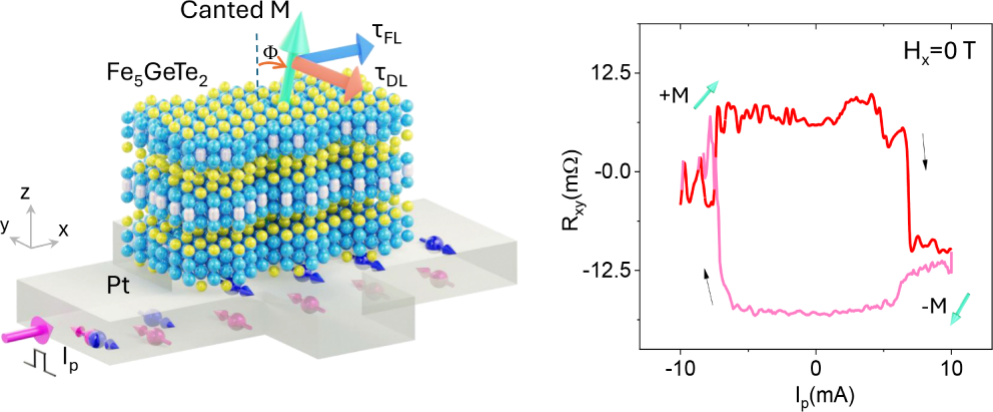

Spin–orbit torque (SOT) magnetization switching is crucial for next-generation energy-efficient spintronic technologies. The recent discovery of van der Waals (vdW) magnets holds promise for such SOT phenomena because of their tunable magnetic properties. However, a demonstration of energy-efficient and field-free SOT switching of vdW magnets is required for their potential applications. Here, we demonstrate field-free and deterministic switching using an intrinsic canted vdW magnet Fe_5_GeTe_2_ in a heterostructure with Pt having a larger spin Hall conductivity up to room temperature. Using anomalous Hall electrical detection for magnetization readout, we reveal that field-free deterministic SOT switching in the Fe_5_GeTe_2_/Pt Hall devices can be attributed to the canted magnetic anisotropy of Fe_5_GeTe_2_, originating from its crystal and magnetic structures. Detailed second harmonic Hall measurements exhibit a high spin Hall conductivity σ*_SH_* ∼ 3 × 10^5^ℏ/2*e* Ω^–1^m^–1^ with an SOT effective damping-like field of 0.06 mT per MA/cm^2^. These findings reveal efficient and field-free SOT phenomena in the canted vdW magnet Fe_5_GeTe_2_ up to room temperature and highlight their usefulness in spintronic devices.

## Introduction

Energy-efficient spintronic technologies are expected to provide solutions for next-generation nonvolatile computing architectures.^1,2^ After the successful utilization of giant magnetoresistance (GMR) and tunnel magnetoresistance (TMR) in data storage and spin-transfer-torque (STT) based nonvolatile memory technologies,^3^ an energy-efficient spin–orbit torque (SOT) phenomenon is considered for faster and ultralow power switching of a nanomagnet for memory,^4−6^ logic,^7^ and neuromorphic computing.^8,9^ In a heterostructure of high spin–orbit material and a ferromagnet, a charge current flowing through the spin–orbit material generates a spin current, which exerts SOT to switch the magnetization of the ferromagnet. However, the requirement of high drive current, the needed assistance of an external magnetic field, and the lack of external control for deterministic SOT switching are the fundamental obstacles to technological applications.^10,11^

Recently discovered van der Waals (vdW) magnets are crucial for SOT technology owing to their low dimensionality, tunable magnetic anisotropy with composition and temperature, proximity-induced phenomena, as well as the possibility of voltage-controlled magnetism.^12−15^ This opens enormous prospects for resolving the material challenges of using traditional ultrathin ferromagnetic metal films^6,16^ and artificial heterostructures.^17−20^ Energy-efficient SOT was demonstrated using vdW magnets with perpendicular magnetic anisotropy (PMA) such as Fe_3_GeTe_2_,^21,22^ Fe_3_GaTe_2_,^23,24^ and Cr_2_Ge_2_Te_6_^25^ in heterostructure with conventional heavy metal Pt^21,22^ or topological insulators (TIs).^26,27^ However, the SOT devices of vdW magnets with strong PMA in heterostructure with Pt or TIs provide an in-plane current-induced spin polarization and need an external in-plane magnetic field to break the symmetry for deterministic magnetization switching.^16^ Recently, field-free deterministic SOT switching of vdW magnets with PMA (Fe_3_GeTe_2_ and Fe_3_GaTe_2_) has been demonstrated in heterostructures with WTe_2_ and TaIrTe_4_ using their out-of-plane SOT component.^28−34^ However, this requires a higher current density for magnetization switching as the spin Hall conductivities of WTe_2_^35,36^ and TaIrTe_4_^32,33^ are limited to σ_*s,z*_∼10^3^−10^4^ ℏ/2*e*Ω^–1^m^–1^, which is orders of magnitude lower than Pt with σ_s,y_∼10^5^ ℏ/2*e*Ω^–1^m^–1^.^21,22,37^ Therefore, practical SOT technologies require energy-efficient and field-free deterministic magnetization switching of vdW magnets in a heterostructure with spin–orbit materials having a larger spin Hall conductivity.

Here, we demonstrate field-free deterministic magnetization switching of an intrinsic canted vdW magnet Fe_5_GeTe_2_ in a heterostructure with Pt having a larger in-plane spin Hall conductivity. Using the anomalous Hall effect (AHE) electrical readout to detect the magnetization change, we demonstrate current-induced magnetization switching up to room temperature without the need for an external magnetic field. Such observed field-free SOT switching is attributed to the canted magnetic anisotropy originating from the crystal and magnetic structure of Fe_5_GeTe_2_.^38^ Furthermore, second harmonic Hall measurements and analysis show a large spin Hall conductivity σ_*SH*_ ∼ 3 × 10^5^ ℏ/2*e* Ω^–1^ m^–1^ and pulse current induced magnetization switching experiments exhibit the low power consumption P ∼ 10^16^ W/m^3^ for the field-free SOT switching.

## Results

### Canted Magnetization of Fe_5_GeTe_2_

**Figure 1:**
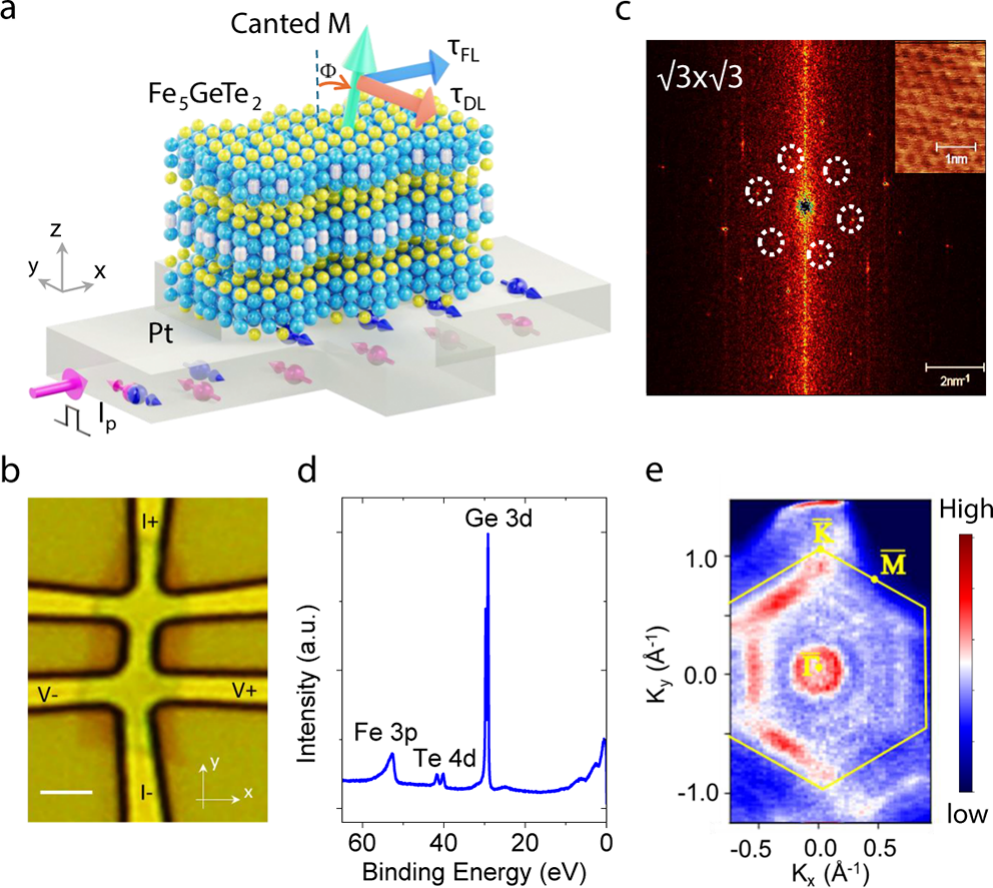
Spin–orbit torque device of Fe_5_GeTe_2_/Pt heterostructure and characterization of Fe_5_GeTe_2_. (a) Schematic of the Fe_5_GeTe_2_/Pt heterostructure Hall-bar device for observation of SOT-induced magnetization switching. The pulsed charge current I_P_ applied to Pt generates a spin current J_s_ due to the spin Hall effect inducing a damping-like torque (τ_DL_) and filed-like torque (τ_FL_) exerted on the magnetic moment M of Fe_5_GeTe_2_. (b) Optical microscope image and measurement geometry of the Fe_5_GeTe_2_/Pt Hall-bar device used for AHE and SOT experiments. (c) Fast Fourier transform (FFT) image of the STM topography of Fe_5_GeTe_2_ (inset showing the higher resolution STM image) measured at room temperature mapping with 1 V and 200 pA. (d) XPS of the Fe_5_GeTe_2_ shows sharp peaks of Fe, Te, and Ge, respectively, probed at 160 eV at 260 K. (e) ARPES density mapping at the Fermi surface as a function of K_x_ and K_y_ in the Fe_5_GeTe_2_ system taken at 20 K with photon energy 138 eV.

Fe_5_GeTe_2_ has gathered increasing attention because of the manifestation of magnetic order above room temperature with *T*_c_ > 300 K and its canted magnetization.^38−41^ Specifically, the canted magnetic properties are established in Fe_5_GeTe_2_/graphene spin-valve devices using Hanle spin precession measurements to probe different spin components.^38^ Such a canted magnetization in Fe_5_GeTe_2_ can provide a possible field-free SOT-induced magnetization switching phenomenon using a larger in-plane spin Hall conductivity of Pt. The schematic and microscope picture of the Fe_5_GeTe_2_/Pt Hall-bar device used for SOT experiments are shown in Figure 1a,b (see more details on the device fabrication in Methods, Figure S1 and Table S1). In a SOT experiment, the application of a series of DC pulse currents I_p_ along the x-direction through the spin–orbit material (Pt), generates an orthogonal spin current J_s_ with spin polarization *s*_y_ along the y-axis due to the spin Hall effect. The generated spin current in Pt exerts SOTs (field-like *τ*_*FL*_ and damping-like *τ*_*DL*_ torques) to switch the magnetization of Fe_5_GeTe_2_. Specifically, a field-like torque, *τ*_*FL*_ ∼ *M* × *s*, precesses *M* about the exchange field created by spin polarization,^42,43^ while a damping-like torque *τ*_*DL*_ ∼ *M* × (*M* × *s*) rotates magnetic moment *M* toward the direction of spin polarization.

To evaluate the quality of the Fe_5_GeTe_2_ crystal, we conducted a scanning tunneling microscopy (STM) analysis of bulk crystal after cleaving the surface in ultrahigh vacuum (Figure 1c). This imaging technique allowed us to resolve the crystal’s atomic arrangement, verifying a √3 × √3 atomic structure.^38^ We carried out an X-ray photoelectron spectroscopy (XPS) measurement (Figure 1d) to examine the elemental compositions. The XPS data confirmed the presence of Fe, Ge, and Te, with no indication of doping from foreign elements or contamination by impurities. Furthermore, Figure 1e presents the Fermi surface map using angle-resolved photoelectron spectroscopy (ARPES), showing distinct band features that reveal a 6-fold symmetry corresponding to the hexagonal Brillouin zone. This observation further supports the high structural quality of the Fe_5_GeTe_2_ crystals.^38^

**Figure 2:**
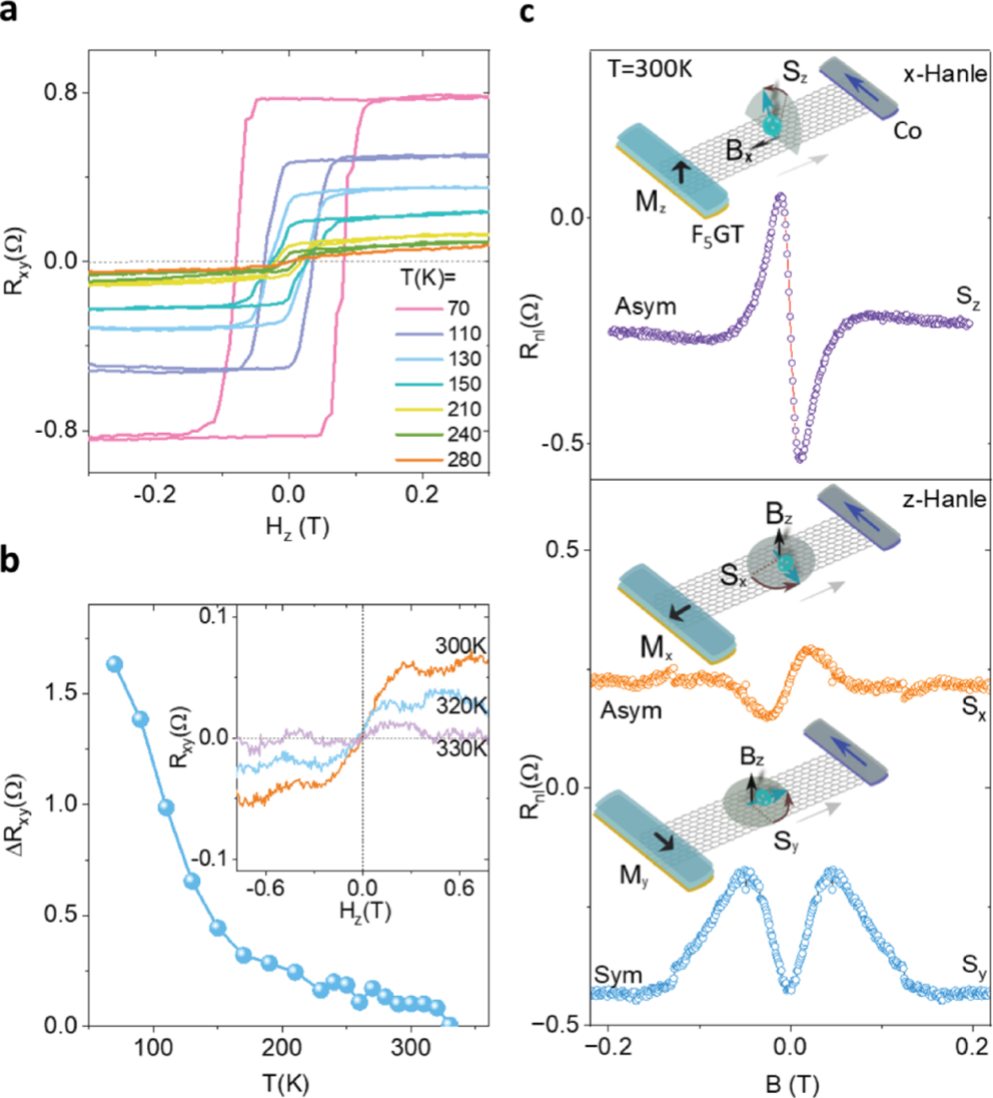
Magnetic properties of Fe_5_GeTe_2_ nanolayers with canted magnetization. (a) AHE resistance R_xy_ as a function of the perpendicular magnetic field H_z_ at different temperatures for a representative Fe_5_GeTe_2_/Pt Hall device. (b) The temperature dependence of the AHE magnitude ΔR_xy_. The inset shows the AHE curves above room temperature. (c) Spin injection experiments with Fe_5_GeTe_2_/graphene spin-valve device. The canted magnetization of Fe_5_GeTe_2_ nanoflake at room temperature results in the observation of x-Hanle and z-Hanle spin precession signals with the asymmetric (Asym) and symmetric (Sym) components. The insets are the schematics for the corresponding measurements and the spin precession dynamics.

To probe the magnetic properties of the Fe_5_GeTe_2_ nanolayer flake, anomalous Hall effect (AHE) measurements were carried out. The transverse magnetoresistance R_xy_ = V_xy_/I_dc_ is probed as a function of the out-of-plane magnetic field H_*z*_. The presence of long-range magnetic order in Fe_5_GeTe_2_ makes it possible to observe the AHE signal, i.e., R_xy_ is proportional to the component magnetization M_z_. Temperature dependence of the AHE signal (Figure 2a,b) shows a decrease in both the magnitude (ΔR_xy_) and the coercive field (H_c_) at higher temperatures. The AHE hysteresis loops measured at low temperatures show a clear magnetic remanence in Fe_5_GeTe_2_. At higher temperatures, the remanence disappears, and the hysteresis loop vanishes; nevertheless, the nonlinearity of the AHE signal persists above room temperature up to 330 K.

To prove the canted magnetism of Fe_5_GeTe_2_, we adopted the spin injection/detection method in Fe_5_GeTe_2_/graphene nonlocal spin valve device.^31^ By performing the x-Hanle and z-Hanle measurements with magnetic field sweeps along x and z directions (Figure 2c), we observed that the x-Hanle signal shows only the asymmetric component and the z-Hanle signal shows both the asymmetric and symmetric components. These observations suggest the coexistence of different spin components (S_x_, S_y_, and S_z_) and canted magnetism with Φ = 13.3° ± 0.5° of the Fe_5_GeTe_2_ nanoflake at room temperature (see detailed analysis in Note S1, Figures S2 and S3).^38^

### Field-Free Deterministic Spin–Orbit Torque Magnetization Switching in Fe_5_GeTe_2_/Pt Heterostructure

**Figure 3:**
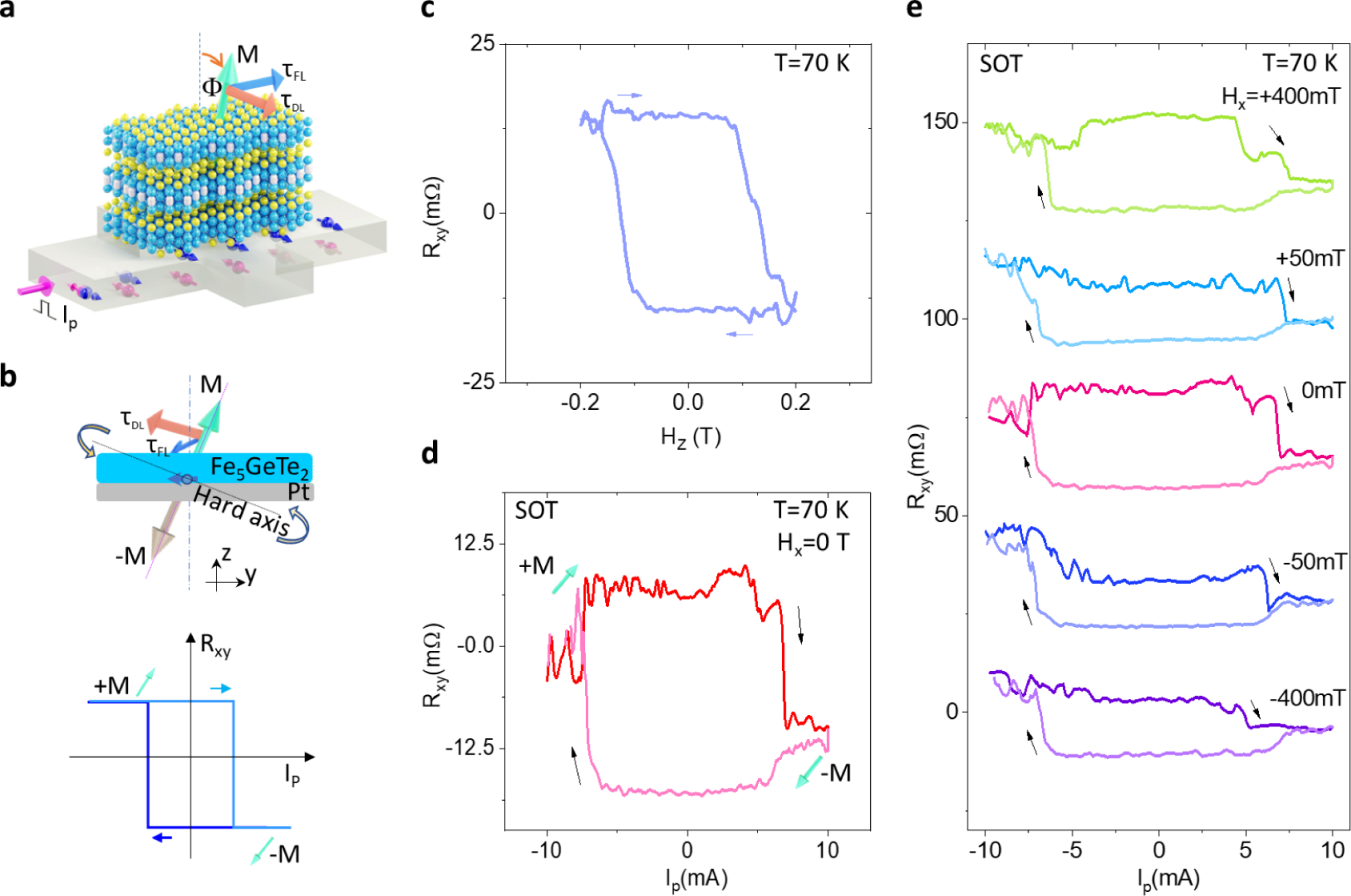
Field-free deterministic SOT magnetization switching of Fe_5_GeTe_2_ with canted magnetization. (a) Schematic of the Fe_5_GeTe_2_/Pt heterostructure with canted magnetic (M) configuration and SOT components (field-like τ_FL_ and damping-like τ_DL_ torques). (b) Possible damping-like and filed-like SOT components with the canted magnetization anisotropy of Fe_5_GeTe_2_ and the expected SOT switching behavior. (c) The AHE signal measured in Fe_5_GeTe_2_/Pt heterostructure in Dev 1 at 70 K. (d) SOT-induced magnetization switching without any external magnetic field H_x_, measured with pulse current I_p_ with a duration of 200 μs and reads current I_r_ of 50 μA. (e) SOT-induced magnetization switching for different external in-plane magnetic fields ±H_x_.

Traditionally, for a strictly perpendicular magnetization, the *τ*_*DL*_ only pulls *M* to the in-plane orientation, and the final magnetization state after the removal of the current can have a random magnetization unless an external field H_x_ is applied to generate an additional field torque allowing for deterministic switching.^16^ However, for a canted magnetization orientation (Figure 3a), the final magnetic state can still be deterministic (from ±*M* to ∓*M)* without an external field due to the geometrical symmetry breaking (Figure 3b).^17^

First, we present SOT-induced magnetization switching of nanolayer Fe_5_GeTe_2_/Pt heterostructure measured at 70 K (Device schematics in Figure 3a). Figure 3c shows the AHE signal of the Fe_5_GeTe_2_/Pt heterostructure Hall-bar device (Dev 1) with H_z_ sweep at 70 K. For the SOT-induced magnetization switching experiments, the Hall resistance R_xy_ = V_xy_/I_r_ was recorded after each pulse current I_p_ applied with a dwell time of 200 μs, where I_r_ is the reading current (50 μA). To be noted, in comparison to only Fe_5_GeTe_2_ measured before, in the Fe_5_GeTe_2_/Pt bilayer devices, the AHE signal is smaller because the contacts are made on Pt and a partial current flow through Fe_5_GeTe_2_. The hysteretic AHE signals with the pulse current swept between ±10 mA (Figure 3d) are observed without the application of any magnetic field (H = 0), which corresponds to a critical switching current density J_sw_ of 3.2 × 10^7^ A/cm^2^. The magnitude of the AHE loops as a function of bias current and magnetic fields are comparable, suggesting a complete switching of the magnetic moment. Such deterministic field-free SOT-induced magnetization switching in Fe_5_GeTe_2_/Pt heterostructure devices can be explained by its canted perpendicular magnetization of Fe_5_GeTe_2_, where the magnetic hard axis is tilted away from the sample plane. A large pulse current-induced damping-like torque *τ*_*DL*_ is needed to overcome the hard magnetization axis and switch to the other direction deterministically. To be noted, the switching direction (chirality) is determined by the canted direction of magnetization and a pulse current sweep would make it possible to switch the magnetic moment between ±*M*, that is, the readout AHE signal R_xy_ (∝ M_z_) shows a bistable state vs I_p_ (Figure 3b). Interestingly, the in-plane external field (H_x_) dependence measurements show that the sign of the SOT switching direction remains unchanged (Figure 3e), suggesting that the applied field does not play a major role in the symmetry breaking for the deterministic switching. Conventionally, the SOT chirality is determined by the external field for a perpendicular magnet, which favors the opposite switching directions with the positive and negative in-plane magnetic fields.^16^ However, in the canted magnetic moment scenario, the in-plane spin ±s_y_ induced damping-like torque solely determines the switching from +*M* to −*M* and vice versa without an external field.

### 2^nd^ Harmonic Hall Spin–Orbit Torque Measurements in Fe_5_GeTe_2_/Pt Heterostructure

**Figure 4:**
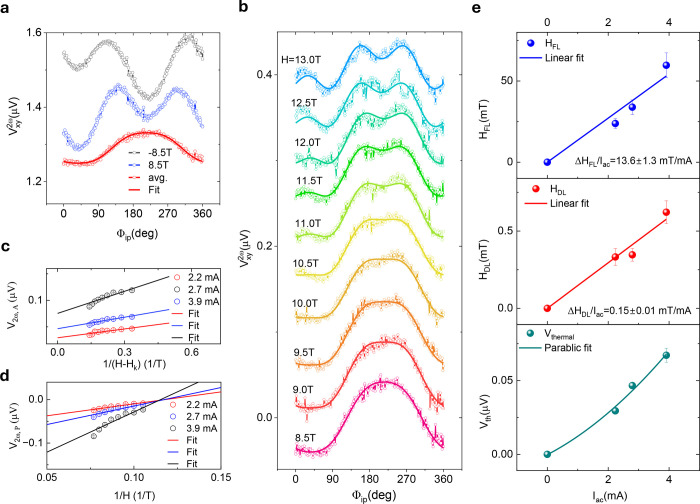
Second harmonic Hall measurements of the SOT effective fields in Fe_5_GeTe_2_/Pt heterostructure. (a) In-plane angle dependence of the 2^nd^ harmonic signals at H = ±8.5 T and the averaged signal (avg.) for ±H in Dev 2 at 120 K. The solid curve is the fitting result with eq 1. (b) Averaged second harmonic signals as a function of the in-plane angle Φ_ip_ at different external fields H. The solid curves are the fitting results with eq 1. (c,d) Extracted components V_2ω,A_ and V_2ω,P_ with different bias currents as a function of the 1/H and 1/(H – H_K_), respectively. The linear solid curves are the fitting results with eqs 2 and 3. (e) Applied bias dependence of the extracted field-like (H_FL_), damping-like (H_DL_) and thermal contribution (V_th_) components.

To quantify the SOT effective damping-like field H_DL_, field-like field H_FL_, and the SOT efficiency quantified by the spin Hall conductivity σ_SH_, we performed the harmonic Hall measurements. The second harmonic Hall signal  as a function of the in-plane rotation angle Φ_ip_ can be expressed,^44−46^
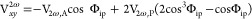
1where
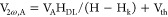
2

and
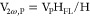
3

with AHE signal V_A_, the effective perpendicular magnetic anisotropy field H_K_, thermal contribution V_th_, and planar Hall effect (PHE) signal V_P_ (see details in Figure S4). Figure 4a shows the measured raw data of the in-plane dependence of the second harmonic signals at both H = ±8.5 T and the averaged result V_avg_ = [V_2ω_(8.5 T) – V_2ω_(−8.5 T)]/2. This procedure rules out any other magnetoresistance signal present in the Fe_5_GeTe_2_/Pt heterostructures. A detailed analysis of the data was presented in Note S2 and Figure S4. The averaged and fitting results at all fields are shown in Figure 4b. A further fitting of the extracted V_2ω,A_ vs 1/(H – H_k_) and V_2ω,P_ vs 1/H are performed to obtain H_DL_, V_th_, and H_FL_ using eq 2 and eq 3, respectively (Figure 4c,d). The applied bias current dependence of the extracted parameters offers more precise results with a linear and parabolic fitting for H_DL(FL)_ and V_th_, respectively (Figure 4e). By using a parallel resistor model to estimate the current flowing through the Pt layer, we obtain ΔH_DL_/J_ac_ = 0.06 ± 0.01 mT per MA/cm^2^ and ΔH_FL_/J_ac_ = 8.64 ± 0.51 mT per MA/cm^2^. The thermal contribution to the harmonic signal V_th_ ∼ I^2^, suggests a vertical thermal gradient ∇T_z_ at Fe_5_GeTe_2_/Pt/SiO_2_/Si, generating a V_th_ ∼ M_x_ × ∇T_z_.^44^

### Spin–Orbit Torque Magnetization Switching in Fe_5_GeTe_2_/Pt Heterostructure at Room Temperature

**Figure 5:**
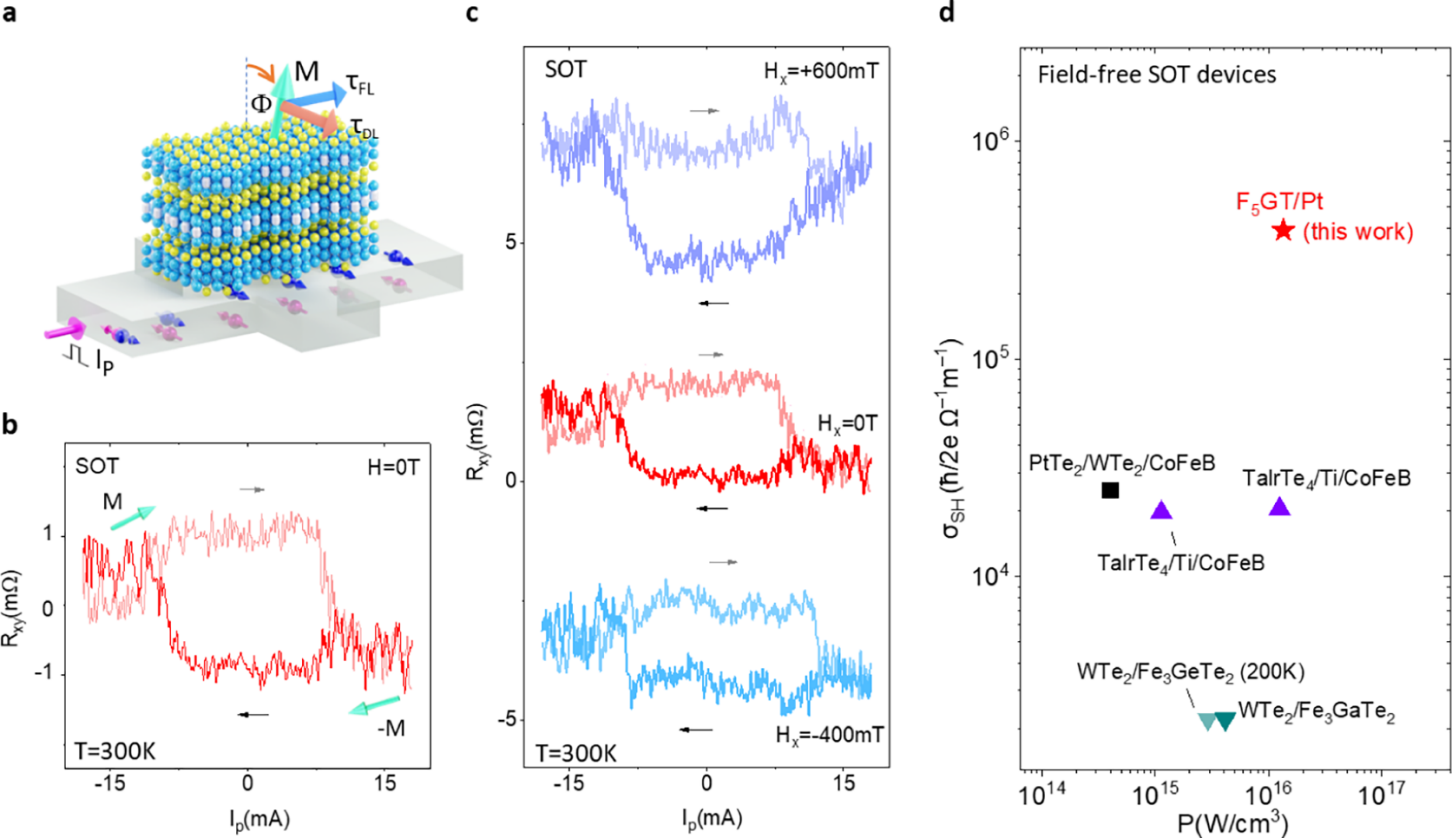
Room temperature field-free deterministic SOT-induced magnetization switching of Fe_5_GeTe_2_. (a) Schematic of the Fe_5_GeTe_2_/Pt SOT device with canted magnetic moment M of Fe_5_GeTe_2_. (b) The SOT-induced magnetization switching without an external magnetic field H_x_ at room temperature. (c) The SOT-induced magnetization switching with an external magnetic field H_x_. The measurements were performed in Dev 3. (d)State-of-the-art of representative field-free SOT devices showing spin Hall conductivity σ_SH_ = θ_SH_σ_c_ of related spin–orbit materials as a function of the power density p = (J_sw_^2^/σ_c_) for the magnetization switching (WTe_2_/Fe_3_GeTe_2_;^28^ WTe_2_/Fe_3_GaTe_2_;^29^ TaIrTe_4_/Ti/CoFeB;^31,32^ PtTe_2_/WTe_2_/CoFeB^33^).

Next, we investigate the magnetization switching of Fe_5_GeTe_2_/Pt heterostructure at room temperature (as shown schematically in Figure 5a). As discussed earlier, the AHE signal with a clear remanence with an in-plane field sweep confirms a canted magnetization at room temperature. Similar to our low-temperature measurements, the SOT magnetization switching measurements of the Fe_5_GeTe_2_/Pt bilayer structure show deterministic switching between two magnetic states by a pulse current induced in-plane damping-like torque, with a critical switching current density J_sw_ of 1.9 × 10^7^ A/cm^2^ with no clear dependence on the direction of the external magnetic field (Figure 5b and Figure 5c). Such room temperature deterministic switching has also been observed in other devices (see Figure S5).

## Discussion

The canted magnetic anisotropy of Fe_5_GeTe_2_ is attributed to the √3 × √3 atomic crystal structure that exists due to Fe vacancies in Fe_5_GeTe_2_,^38,47^ agreeing well with our observations of the SOT experiments and magnetic properties in Fe_5_GeTe_2_.^48^ The in-plane external field (H_x_) dependence measurements show that the sign of the SOT switching direction remains unchanged, confirming that the applied field does not play a major role in the symmetry breaking for the deterministic switching. Instead, the observation of field-free deterministic magnetization switching of Fe_5_GeTe_2_/Pt heterostructure can be explained by considering the canted-perpendicular magnetization of Fe_5_GeTe_2_.

To be noted, the calculation of SOT-induced switching efficiency η_sw_ based on the pulse-induced magnetization switching is usually overestimated.^49^ The current-induced torques only need to overcome the depinning or nucleation barrier with the assistance of Joule heating, which is much smaller than the anisotropy barrier, so that the switching efficiency η_sw_ is usually much larger than the spin Hall angle θ_SH_.^49^ So, we calculate the spin Hall conductivity σ_SH_ = θ_SH_σ_c_, (σ_c_ is the charge conductivity), which considers both spin-charge conversion efficiency θ_SH_ and the conductivity of the spin–orbit materials (SOMs) to minimize power consumption. This is a universal figure of merit to characterize the SOT performance.^50^ Using the values obtained for our devices (θ_SH_ > 0.12 and σ_c_ = 2.5 × 10^6^ S/m, we estimate σ_SH_ = (3.0 ∼ 7.5) × 10^5^ ℏ/2*e* Ω^–1^ m^–1^, which is attributed to the transparent Fe_5_GeTe_2_/Pt interface^21,22^ (see more details in Table S2). Figure 5d shows the state-of-the-art of representative field-free SOT devices, showing spin Hall conductivity σ_SH_ of related spin–orbit materials as a function of the power density P of the representative field-free SOT devices. Our work on Fe_5_GeTe_2_/Pt heterostructure is among the best field-free SOT devices in terms of spin Hall conductivity σ_SH_ and the power consumption P. Furthermore, observing field-free and deterministic SOT magnetization switching of vdW magnets up to room temperature using conventional spin Hall materials such as Pt can have advantages over other systems.

## Conclusions

In summary, we demonstrate an external magnetic field-free deterministic magnetization switching in Fe_5_GeTe_2_/Pt heterostructures up to room temperature. The observation of such SOT-induced magnetization switching is enabled by the canted magnetization of Fe_5_GeTe_2_. Detailed second harmonic Hall measurements and analysis show a large SOT-induced magnetization switching efficiency with spin Hall conductivity σ_SH_ ∼ 3 × 10^5^ ℏ/2*e* Ω^–1^ m^–1^. The SOT devices using Fe_5_GeTe_2_/Pt heterostructures highlight the potential of using canted vdW magnetic material and conventional spin–orbit materials with large spin Hall conductivity. In the future, all-vdW heterostructures of Fe_5_GeTe_2_ can also be used together with topological materials having even larger charge-spin conversion properties. Encouragingly, the *T*_c_ of vdW magnetic materials can be further enhanced much beyond room temperature, and their magnetic properties can be engineered with alloys of Co^51−53^ and Ni.^54,55^ Combining spin–orbit materials with large charge-spin conversion efficiency and vdW magnets with faster magnetization dynamics can be envisioned as new building blocks for future energy-efficient spintronic applications.

## Methods/Experimental

Device Fabrication: The Pt layer (10 nm) is first globally deposited on a SiO_2_/Si substrate with a Ti seed layer (2 nm). FGT flakes are exfoliated and transferred onto the Pt layer inside a Nitrogen gas glovebox. This is followed by patterning of the Pt and FGT films using electron beam lithography and Ar-ion plasma etching to define the Hall bar geometry.

Measurements: Transport measurements in Fe_5_GeTe_2_ and Fe_5_GeTe_2_/Pt devices were performed in a vacuum cryostat with a magnetic field. The electronic measurements were carried out using the current source Keithley 6221 and nanometer 2182A. To monitor the longitudinal and Hall resistances, Keithley 2182A nanovoltmeters were used. For the current-induced switching measurements in the F_5_GT/Pt devices, Keithley 2182A nanovoltmeters were used to monitor the response of the Hall resistances, whereas a Keithley 6221 AC source was used with a pulse current of 100 μs through the device. The harmonic measurement was performed using Lock in SR830 to measure in-phase first and out-of-phase second harmonic voltages with *f* = 213.34 Hz, respectively. The second harmonic measurements in the high magnetic field range were carried out in the Quantum Design cryogen-free PPMS DynaCool system with an external electronic connection to Lock in SR830 to measure the first and second harmonic voltages.

XPS, ARPES, STM: XPS of the single crystal was probed at photon energy of 160 eV at 260 K. ARPES: The angle-resolved photoelectron spectroscopy (ARPES) measurements were performed at the MAX IV Laboratory Bloch beamline with high energy, angular, and spatial resolution (15 meV, < 0.15 degrees, 10 μm × 15 μm). The measurements were done at 20 K at 138 eV photon energy. Single crystals of Fe_5_GeTe_2_ were cleaved in a high vacuum better than 8 × 10^–11^ mbar. A deflector-type hemispherical analyzer from Scienta Omicron was used. STM: The scanning tunneling microscopy (STM) measurements were done at 300 K using the VT-XA model from Scienta Omicron.

## Data Availability

The data that support the findings of this study are available from the corresponding authors on reasonable request.

## Supporting Information Available

Additional information and figures regarding the summary of the devices/samples and the corresponding measurement techniques; canted magnetic anisotropy of Fe_5_GeTe_2_; SOT efficiency of the Fe_5_GeTe_2_/Pt heterostructure; SOT parameters of the measured Fe_5_GeTe_2_/Pt devices (PDF)
