# Supplementary material for: Field-Free Spin–Orbit Torque Switching of Canted van der Waals Magnets
Source: ACS Nano. 2025 Mar 31;19(14):13817–24. doi: 10.1021/acsnano.4c16826 (PMC12004925; doi:10.1021/acsnano.4c16826)
Supplement: Supplementary file 1 — nn4c16826_si_001.pdf [file nn4c16826_si_001.pdf]

Supplementary information

Field-Free Spin-Orbit Torque Switching of Canted van der Waals Magnet

Bing Zhao[1*], Lalit Pandey[1,6], Khadiza Ali[1,2], Erdi Wang[1], Craig M. Polley[2], Balasubramanian Thiagarajan[2], Peter Makk[3,4], Marcos H. D. Guimarães[5], Saroj Prasad Dash[1,6,7*]

[1]Department of Microtechnology and Nanoscience, Chalmers University of Technology, SE-41296, Göteborg, Sweden

[2]MAX IV Laboratory, Lund University, Lund SE-221 00, Sweden

[3]Department of Physics, Budapest University of Technology and Economics, H-1111 Budapest, Hungary.

[4]MTA-BME Correlated van der Waals Structures Momentum Research Group, Műegyetem rkp. 3., H-1111 Budapest, Hungary

[5]Zernike Institute for Advanced Materials, University of Groningen, Groningen, The Netherlands

[6]Wallenberg Initiative Materials Science for Sustainability, Department of Microtechnology and Nanoscience, Chalmers University of Technology, SE-41296, Göteborg, Sweden.

[7]Graphene Center, Chalmers University of Technology, SE-41296, Göteborg, Sweden.

Supplementary Table S1. Summary of the devices/samples and the corresponding measurement techniques.

Measurments	Samples/structures	Note
STM topography	bulk Fe_5GeTe_2 sample	Fig.1c
XPS	bulk Fe_5GeTe_2 sample	Fig .1d
ARPES	bulk Fe_5GeTe_2 sample	Fig .1e
AHE signals	Fe_5GeTe_2 nanolayer Hallbar	Fig. 2a
Hanle measurments	Fe_5GeTe_2/graphene/Co nonlocal spin valve	Fig. 2c and Supplementary Fig .S2
Pulse current induced SOT	Fe_5GeTe_2/Pt hetrostructure Hallbar device	Fig .3 (Dev 1); Fig .5 (Dev 3); Supplementary Fig .S4 (Dev 4)
Second-harmonic Hall measurments	Fe_5GeTe_2/Pt hetrostructure Hallbar device	Fig .4 and Supplementary Fig .S3 (Dev 2)
AFM thickness profile	Fe_5GeTe_2/Pt hetrostructure	Supplementary Fig .S1 (Dev 1)

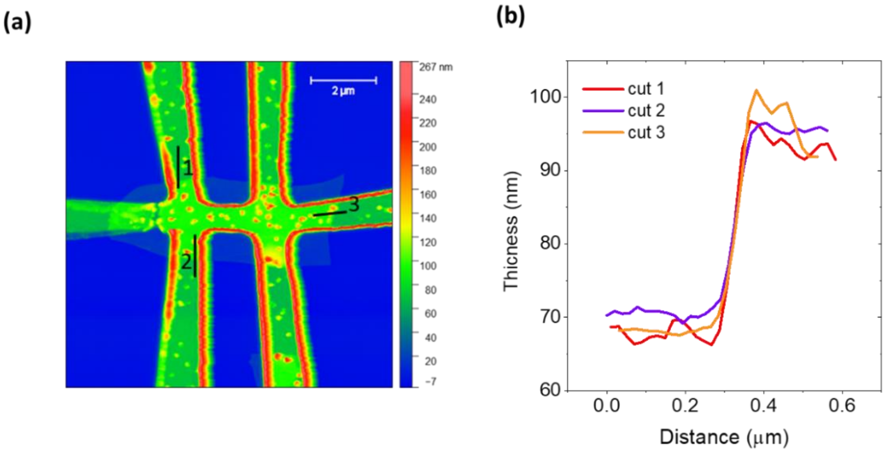

Supplementary Figure S1. Atomic force macroscopic image of Fe_5GeTe_2/Pt heterostructure. (a) *Atomic force microscopy (AFM) mapping of the Fe_5GeTe_2/Pt of Dev 1.* ***(b)*** *Thickness profile of the cuts indicated in a) with a thickness of ~25 nm.*

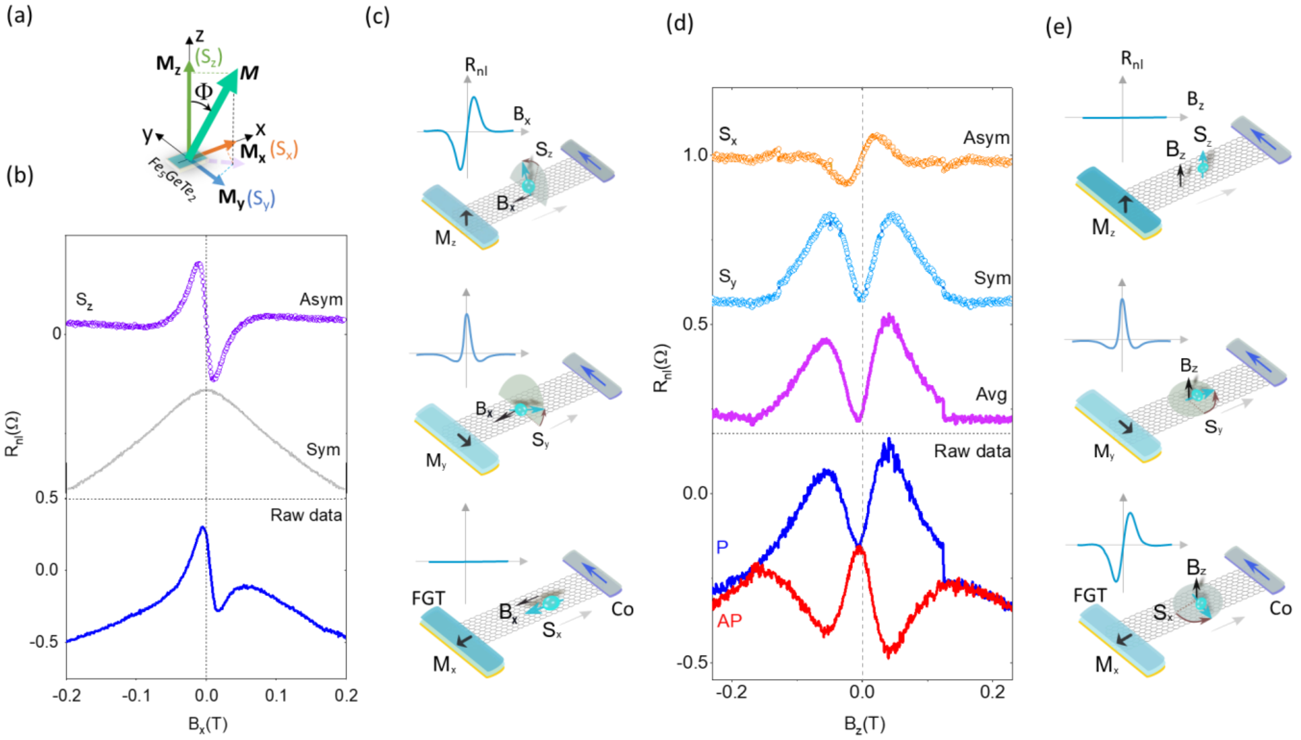

Figure S2. X(z)-axis Hanle signals in Fe_5GeTe_2-graphene spin valve. (a) *Schematic of the magnetic moment configuration in Fe_5GeTe_2, where M represents the total magnetic moment of Fe_5GeTe_2 with components (M_x, M_y, M_z) along the x-and y-axis. $S_{x(y,z)}$ is the injected spins from M with components $M_{x(y,z)}$, along the x(y,z)-axis, respectively. Φ is the angle between M and the z-axis.* ***(b)*** *Measured x-Hanle spin precession signal as a function of B_x and the decomposed symmetric (Sym) and asymmetric (Asym) components.* ***(d)*** *Measured z-Hanle signal and the decomposed Sym and Asym components from averaged R_{avg}, where $R_{avg}=[R_{nl}(P)-R_{nl}(AP)]/2$.* ***(c, e)*** *Schematics of x(z)-Hanle measurement geometries and expected lineshape of the spin precession signals for all the possible Fe_5GeTe_2 magnetization scenarios (M_x, M_y, and M_z).*

Supplementary Note S1. Canted magnetic anisotropy of Fe_5GeTe_2

(1) The Hanle measurement to identify magnetic anisotropy,[1] takes advantage of the spin injection from the van der Waal magnet with all possible spin polarizations, demonstrating a reliable way to identify the canted magnetic anisotropy with nanolayer samples. To prove the canted magnetization of Fe_5GeTe_2, we perform measurements on the Fe_5GeTe_2/graphene nonlocal spin valve devices.[1] As shown in the schematics (Fig. S2a), a possible canted magnetization of M has the projection along all the axes. Correspondingly, the Fe_5GeTe_2 can inject a spin current with different spin polarization $S_{x(y,z)}$ components along the magnetic moment. By performing x (z)- Hanle measurements, we can extract the specific spin components as shown in Fig. S2c (Fig. S2e). The x-Hanle signal in Fig. S2b combines the symmetric (Sym) and asymmetric (Asym) components. The asymmetric Hanle signal is due to the S_z spin injection. The symmetric component here is not a Hanle spin precession signal, but just a background signal originating from the rotation of the magnetic moment at a larger field. Similarly, the z-Hanle signals in Fig. S2d provide evidence of spin polarization in the xy-plane, where we extract the existence of the S_x and S_y. Therefore, it is the canted magnetic moment in the Fe_5GeTe_2 nanoflake that presents the coexistence of all the three spin directions $S_{x(y,z)}$, corresponding to the $M_{x(y,z)}$, ie. a canted magnetization in Fe_5GeTe_2 with Φ=13.3°±0.5°.

(2) Origin of the canted magnetism - Two mechanisms work together to induce canted magnetic anisotropy. First, exchange interactions between spins can introduce canted spin texture along the b-axis,[1–3] where spins may have a specific averaged angle Φ dependent on the Fe sites (see Fig. 3). Second, canting also comes from the magnetocrystalline anisotropy energy (MAE), then spins will tend to get canted, but remain relatively parallel to each other due to long-range magnetic interaction. Therefore, both factors decide the canted magnetic anisotropy in a real magnetic system. In short, these results suggest that the tilted anisotropy is a universal property for FGT. The tilted easy magnetization axis prefers to align to the b-axis of the crystal direction[1–3].

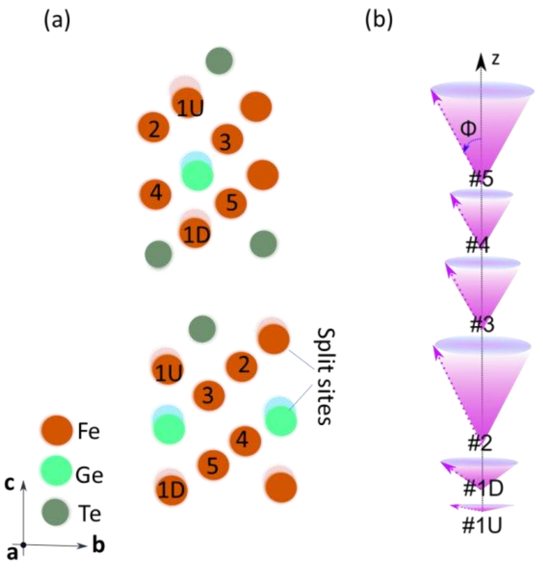

Supplementary Figure S3. Canted magnetism in Fe_5GeTe_2. (a) *Crystal structure of Fe_5GeTe_2.* ***(b)*** *Averaged Fe canted angle at different Fe positions. The dashed arrow shows the magnetic moments and its canted angle Φ. The length of the arrows represents the strength of the angular momentum in each Fe site.*[1–3]

Supplementary Note S2. SOT efficiency of the Fe_5GeTe_2/Pt heterostructure

Details on the analysis of 2nd harmonic Hall signals – The 2nd harmonic Hall signal $V_{xy}^{2\omega}$ could have other contributions if the sample is not restricted to the magnetic field plane with a small angle θ< 5 degree (see below). So that we can rewrite the 2nd harmonic Hall signal.[4]

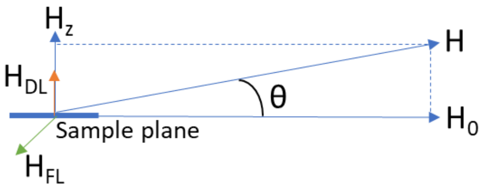

$V_{xy0}^{2\omega}$=-$V_{2\omega\_A}\cos\Phi_{ip}$ + $2V_{2\omega\_P}(2\cos^3\Phi_{ip}-\cos\Phi_{ip})+H\theta V_A/[2(H-H_k)]+c$; (Eq. S1)

In the measurement, we perform +/-H related angle dependence of $V_{xy0}^{2\omega}$, so that $[V_{xy0}^{2\omega}(H) - V_{xy0}^{2\omega}(-H)]/2= V_{xy}^{2\omega}$ to obtain Eq. 1 in the main text. Then we can remove some sample angle tilt-related contributions.

SOT efficiency can be evaluated by the equation,[5]

$\xi_{DL}=T_{ini}\theta_{SH}=\frac{2e}{\hbar}\mu_0 M_s t_{FGT}\Delta H_{DL}/J_{ac}$; (Eq. S2)

where e is the electron charge, $\hbar$ is the reduced Plank constant, $\mu_0 M_s \approx 600$ emu/cm^3 is the saturation magnetization of Fe_5GeTe_2[6] (the low-temperature saturation magnetization is assumed to be the same as the one at room temperature), t_{FGT} is the thickness of Fe_5GeTe_2, and $\Delta H_{DL}/J_{ac}$ is the effective damping-like field per switching current density in Pt. T_{ini} (≈1) is the transparency factor of the Fe_5GeTe_2/Pt interface. See detailed results in Table S2.

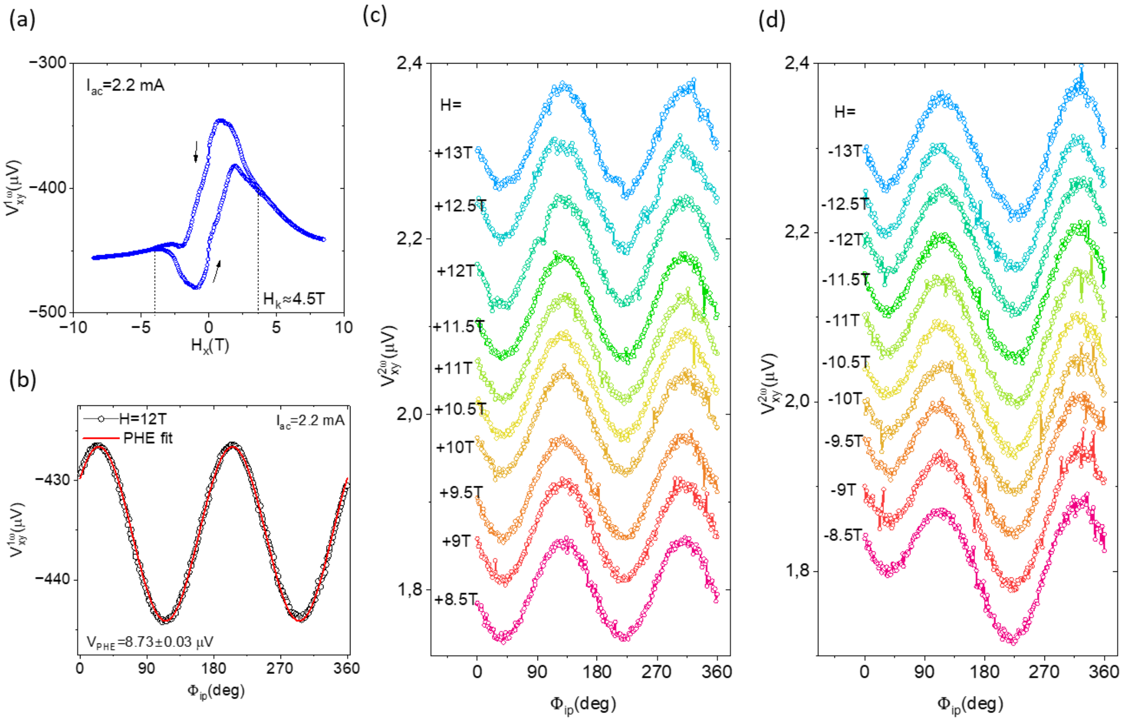

Supplementary Figure S4. First and second harmonic Hall measurements of the SOT effective fields in Fe_5GeTe_2/Pt heterostructure of Dev 2. (a, b) *1st harmonic signals as a function of in-plane field H_x and in-plane angle rotation Φ_{ip}, respectively. The solid curve is the fitting result of ($V_{xy}=V_{PHE}\sin(2\Phi)+c$) to extract V_{PHE}.* ***(c, d)*** *Raw data of the 2nd harmonic signals as a function of the in-plane angle Φ_{ip} at positive and negative fields H, respectively.*

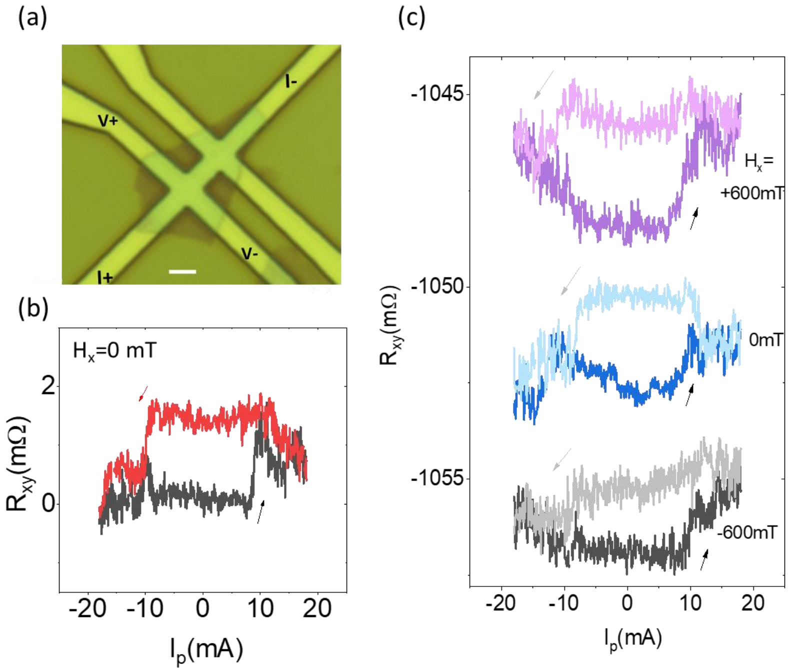

***Supplementary Figure S5. Room temperature magnetization switching by SOT in Fe_5GeTe_2/Pt. (a)** Optical image of the Dev 4 with the measurement configuration. The scale bar is 2 µm. **(b, c)** The SOT magnetization switching curves with and without an external magnetic field.*

Supplementary Table S2. SOT parameters of the measured Fe_5GeTe_2/Pt devices.

The parameters of Fe_5GeTe_2/Pt SOT Hall devices: material thickness t_{Pt}=10 nm, t_{F5GT}. The Fe_5GeTe_2/Pt Hall channel width W; Different SOT characterization methods, such as second-harmonic Hall (SHH) and pulsed-current switching (PCS) at different temperatures T. $\rho_{FGT}=1.5*10^{-6}$ Ωm and $\rho_{Pt}=4*10^{-7}$ Ωm are extracted based on the four-probe method. Ratio $\zeta=I_{pt}/I_{total}$ is calculated by a two-parallel channel model. Pulsed-current induced magnetization switching current density $J_{sw}=\zeta I_{sw}/(Wt_{Pt})$.

Devices	t_{F5GT} (nm)	$\zeta=I_{pt}/I_{tot}$	W (µm)	H_{DL}/J_{ac} (mT per MA/cm²)	H_{FL}/J_{ac} (mT per MA/cm²)	T (K)	ξ_{DL}	J_{sw} (MA/cm²)	Method
Dev 1 (F_5GT-Pt-2-15)	25	0.6	1.3	-	-	70	-	32	PCS
Dev 2 (F_5GT-Pt-5-11)	70	0.348	1.4	0.06	8.64	120	~0.12	-	SHH
Dev 3 (F_5GT-Pt-3-15)	40	0.48	2.5	-	-	300	-	19	PCS
Dev 4 (F_5GT-Pt-2-18)	40	0.48	2.0	-	-	300	-	24	PCS

Reference

(1) Zhao, B.; Ngaloy, R.; Ghosh, S.; Ershadrad, S.; Gupta, R.; Ali, K.; Hoque, A. M.; Karpiak, B.; Khokhriakov, D.; Polley, C.; Thiagarajan, B.; Kalaboukhov, A.; Svedlindh, P.; Sanyal, B.; Dash, S. P. A Room-Temperature Spin-Valve with van Der Waals Ferromagnet Fe5GeTe2/Graphene Heterostructure. *Adv. Mater.* **2023**, *35* (16), 2209113.

(2) Ly, T. T.; Park, J.; Kim, K.; Ahn, H.; Lee, N. J.; Kim, K.; Park, T.; Duvjir, G.; Lam, N. H.; Jang, K.; You, C.; Jo, Y.; Kim, S. K.; Lee, C.; Kim, S.; Kim, J. Direct Observation of Fe-Ge Ordering in Fe5−xGeTe2 Crystals and Resultant Helimagnetism. *Adv. Funct. Mater.* **2021**, *31* (17), 2009758.

(3) Ershadrad, S.; Ghosh, S.; Wang, D.; Kvashnin, Y.; Sanyal, B. Unusual Magnetic Features in Two-Dimensional Fe5GeTe2 Induced by Structural Reconstructions. *J. Phys. Chem. Lett.* **2022**, *13* (22), 4877–4883.

(4) Bose, A.; Schreiber, N. J.; Jain, R.; Shao, D.-F.; Nair, H. P.; Sun, J.; Zhang, X. S.; Muller, D. A.; Tsymbal,

E. Y.; Schlom, D. G.; Ralph, D. C. Tilted Spin Current Generated by the Collinear Antiferromagnet Ruthenium Dioxide. *Nat. Electron.* **2022**, *5* (5), 267–274.

(5) Nguyen, M.-H.; Ralph, D. C.; Buhrman, R. A. Spin Torque Study of the Spin Hall Conductivity and Spin Diffusion Length in Platinum Thin Films with Varying Resistivity. *Phys. Rev. Lett.* **2016**, *116* (12), 126601.

(6) Seo, J.; Kim, D. Y.; An, E. S.; Kim, K.; Kim, G.-Y.; Hwang, S.-Y.; Kim, D. W.; Jang, B. G.; Kim, H.; Eom, G.; Seo, S. Y.; Stania, R.; Muntwiler, M.; Lee, J.; Watanabe, K.; Taniguchi, T.; Jo, Y. J.; Lee, J.; Min, B. Il; Jo, M. H.; Yeom, H. W.; Choi, S.-Y.; Shim, J. H.; Kim, J. S. Nearly Room Temperature Ferromagnetism in a Magnetic Metal-Rich van Der Waals Metal. *Sci. Adv.* **2020**, *6* (3), eaay8912.
